# Prognostic Value of Serum Albumin at Admission for Neurologic Outcome with Targeted Temperature Management after Cardiac Arrest

**DOI:** 10.1155/2019/6132542

**Published:** 2019-09-02

**Authors:** Soo Hyun Kim, Chun Song Youn, Hyo Joon Kim, Seung Pill Choi

**Affiliations:** ^1^Department of Emergency Medicine, Eunpyeong St. Mary's Hospital, College of Medicine, The Catholic University of Korea, Seoul 03312, Republic of Korea; ^2^Department of Emergency Medicine, Seoul St. Mary's Hospital, College of Medicine, The Catholic University of Korea, Seoul 06591, Republic of Korea

## Abstract

**Introduction:**

It is well known that hypoalbuminemia is associated with adverse outcomes in various critical illnesses. However, there are few studies specifically measuring the association between albumin level and neurologic outcomes after CA treated with TTM. The aim of this study was to assess whether serum albumin concentration on admission had prognostic value for OHCA patients treated with TTM.

**Methods:**

We included adult patients aged ≥18 years with nontraumatic OHCA treated with TTM whose serum albumin concentration was available and who were admitted from 2009 to 2016. Serum albumin was measured within 1 h after ROSC, and hypoalbuminemia was defined as admission serum albumin <3.5 g/dl. A good neurologic outcome was defined as a cerebral performance category score of 1 or 2 at 6 months.

**Results:**

A total of 255 patients were eligible for analysis, of whom 106 (41.6%) survived to 6 months; 84 (32.9%) of these patients achieved favorable neurologic outcomes. The mean albumin values were significantly lower in patients with poor neurologic outcomes than the values in those with good neurologic outcomes (3.3 ± 0.6 vs. 3.9 ± 0.4, respectively, *p* < 0.001). After adjusting the crude model, patients in the hypoalbuminemia group were 3.5 times more likely to have poor neurologic outcome than were those in the normal albumin group (OR 3.526, 95% CI 1.388–8.956, *p*=0.008).

**Conclusions:**

Hypoalbuminemia was common after CA, and the serum albumin level at admission was associated with poor neurological outcomes at 6 months after CA in patients treated with TTM.

## 1. Introduction

Cardiac arrest (CA) is a health problem worldwide and is associated with high rates of mortality and morbidity [1]. Although the major pathophysiologic mechanisms after CA have not yet been elucidated, systemic inflammation after ischemic reperfusion injury contributes to hypoxic brain injury, the major leading cause of death after CA [2]. To protect the brain and other critical organs from systemic inflammation, targeted temperature management (TTM) at 32–36 for 24 hrs is considered the standard of care after CA [3–5]. Optimizing cardiopulmonary function and minimizing reperfusion injury should be focused during post-cardiac arrest care [6].

Albumin is the main protein of human plasma, playing several important physiologic roles, including maintenance of plasma oncotic pressure and microvascular integrity and regulating vascular functions, antioxidant activities, and anticoagulant effects [7–9]. The systemic inflammatory response has been associated with decreased synthesis and increased catabolism of albumin [10]. Hypoalbuminemia has been shown to increase blood viscosity and cause endothelial dysfunction [11–13]. It is well known that hypoalbuminemia is associated with adverse outcomes in various critical illnesses, especially cardiovascular disease, stroke, sepsis, and coronary artery bypass grafting [14–18]. However, there are few studies specifically measuring the association between the albumin level and neurologic outcomes after CA with TTM.

Therefore, the aim of this study was to test the hypothesis that the serum albumin level on admission was associated with neurological outcomes of out-of-hospital CA patients treated with TTM. We combined this association with other clinical variables to assess the value of albumin in predicting neurological outcomes.

## 2. Materials and Methods

### 2.1. Study Population

This retrospective analysis using prospectively collected data was conducted at the Seoul St. Mary's Hospital, a tertiary urban teaching hospital, from 2009 to 2016. Our institutional review board approved this study. The requirement for informed consent was waived because of the retrospective nature of this study. All comatose patients who were resuscitated and brought to the emergency department were treated with post-cardiac arrest care protocols including TTM [19]. The inclusion criteria were as follows: older than 18 years of age; resuscitated from out-of-hospital CA and treated with TTM; and having an albumin level measured within 1 hr after ROSC. Patients were excluded if the cardiac arrest was caused by trauma or if TTM was not initiated or was interrupted due to hemodynamic instability, recurrent lethal arrhythmia, or severe bleeding.

### 2.2. Clinical Variables

The study participants' medical records were reviewed. The following demographic and clinical data were collected for each patient: age, sex, preexisting disease (e.g., hypertension, diabetes mellitus, coronary artery disease, renal disease, liver disease, stroke, and malignancy), cause of arrest, witnessed collapse, bystander cardiopulmonary resuscitation (CPR), rhythm initially presenting after arrest, time from collapse to restoration of spontaneous circulation (ROSC), post-ROSC neurologic examinations (e.g., pupillary light reflex (PLR), self-respiration, and Glasgow Coma Scale (GCS)), hemodynamic status, and length of stay. Definitions were based on Utstein-style guidelines [20]. Serum albumin was measured within 1 h after ROSC, and hypoalbuminemia was defined as admission serum albumin <3.5 g/dl.

### 2.3. Outcome

The primary outcome of this study was 6-month neurologic outcome. Neurologic outcome was assessed using the cerebral performance category (CPC) score [21]. The five categories of the CPC are as follows: CPC 1, conscious and alert with good cerebral performance; CPC 2, conscious and alert with moderate cerebral performance; CPC 3, conscious with severe cerebral disability; CPC 4, comatose or in persistent vegetative state; and CPC 5, brain dead, circulation preserved, or death at discharge. Favorable neurologic outcomes were defined as CPC scores of 1 and 2.

### 2.4. Statistical Analysis

We tested the distributions of the continuous variables for normality using visual inspection and the Shapiro–Wilk test. Normally distributed data were expressed as the means and standard deviations using Student's *t*-test. Nonnormally distributed data were assessed using the Mann–Whitney *U* test. Categorical variables were presented as frequency with percentage and were compared using the *χ*^2^ test or Fisher's exact test, as appropriate. Multivariate binary logistic regression analysis was used to assess independent predictors of 6-month poor neurologic outcome. All variables with a significance level <0.1 by univariate analysis were entered into the multivariate logistic regression model to create a crude model. The factors with *p* values < 0.05 on the multivariate logistic regression model were entered into a crude model. We considered factors in the crude model as established risk factors because no confirmed risk factors exist for predicting 6-month poor neurologic outcome. To evaluate the association of serum albumin with 6-month poor neurologic outcome, the serum albumin values were divided into 2 categories using the following cutoff values: <3.5 mg/dl and ≥3.5 mg/dl. Odds ratios (ORs) and 95% confidence intervals (CIs) were calculated with the lowest quartile as the reference. Serum albumin was examined as a continuous variable as well. All statistical analyses were performed using SPSS software, version 17.0 (SPSS, Chicago, IL, USA). Values of *p* < 0.05 were considered statistically significant for all comparisons.

## 3. Results

### 3.1. Characteristics of the Study Population

During the study period, a total of 410 consecutive patients with nontraumatic out-of-hospital cardiac arrest (OHCA) were admitted to our ED. Sixty-six patients did not receive TTM, 7 had no serum albumin data, and 255 were eligible for our analyses, of whom 106 (41.6%) survived to 6 months; 84 (32.9%) achieved favorable neurologic outcomes.

The mean patient age was 54.7 ± 16.2 years, and 180 patients (71%) were male; 107 patients (42.0%) died during their hospital stay, and the mean serum albumin level was 3.5 ± 0.6 mg/dl.  Table 1 presents the characteristics and preresuscitation and postresuscitation data of eligible patients. The hypoalbuminemia group had a higher proportion of older age and history of diabetes or renal disease and had a longer time from collapse to ROSC. The hypoalbuminemia group was less likely to have first documented shockable rhythm and positive results for post-ROSC neurologic examination (e.g., PLR, GCS motor, and self-respiration). The mean arterial pressure immediately after ROSC was significantly lower in the hypoalbuminemia group than that in the normal albumin group. Regarding outcomes, the normal serum albumin group had the highest proportion of favorable neurological outcome and 6-month survival (Table 1).

### 3.2. Univariate and Multivariate Analysis of Factors Associated with 6-Month Neurologic Outcome

The mean albumin values were significantly lower in the poor neurologic outcome group than in the good neurologic outcome group (3.3 ± 0.6 vs. 3.9 ± 0.4, respectively; *p* < 0.001) (Table 2). On univariate analysis, age, history of HTN, diabetes mellitus, renal disease and malignancy, cause of arrest, initial documented rhythm, witnessed collapse, and time from collapse to ROSC, PLR, self-respiration, and GCS motor, mean arterial pressure, and temperature were all significantly associated with 6-month neurologic outcome. Variables with *p* values < 0.1 on univariate analysis were entered into the multivariate logistic regression model to create a crude model, and the factors with *p* values < 0.05 in the multivariate logistic regression model were entered into the crude model. The crude model included the following variables: cardiac etiology, initial rhythm, time from collapse to ROSC, and GCS motor.

After adjusting the crude model, albumin levels still showed an association with 6-month poor neurologic outcome. Patients in the hypoalbuminemia group were 3.5 times more likely to have poor neurologic outcome than were those in the normal albumin group (Model I, Table 3). After adjusting the crude model and history of liver disease, renal disease, and malignancy, this association remained. Furthermore, patients in the hypoalbuminemia group were 2.7 times more likely to have poor neurologic outcome than were those in the normal albumin group (model II, Table 3). When serum album was examined as a continuous variable, there were significant associations with 6-month poor neurologic outcome after adjusting for model I covariates (OR = 0.222, 95% CI = 0.095–0.516) and after model II covariates (OR = 0.275, 95% CI = 0.116–0.651) (Table 3). These findings suggested that there was 77.8% and 72.8% more favorable neurologic outcome for every 1 mg/dl of serum albumin (Table 3).

### 3.3. Prognostic Value of Hypoalbuminemia

**Figure 1:**
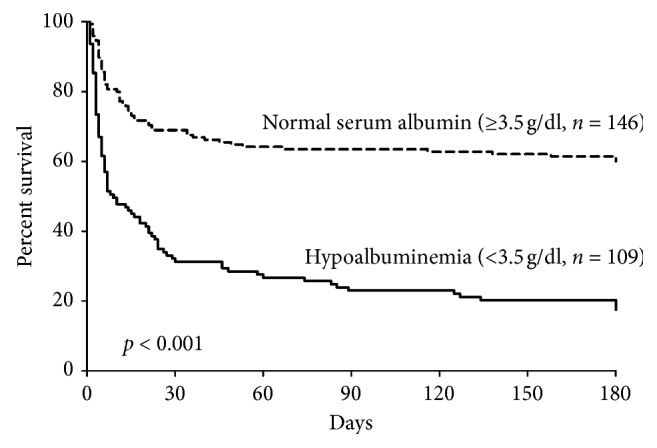
180-day Kaplan–Meier survival analysis over time stratified by serum albumin level.

Kaplan–Meier analysis revealed that the survival rate of patients with hypoalbuminemia was significantly lower than in those with serum albumin levels at or above 3.5 g/dL (*p* < 0.001; Figure 1).

**Figure 2:**
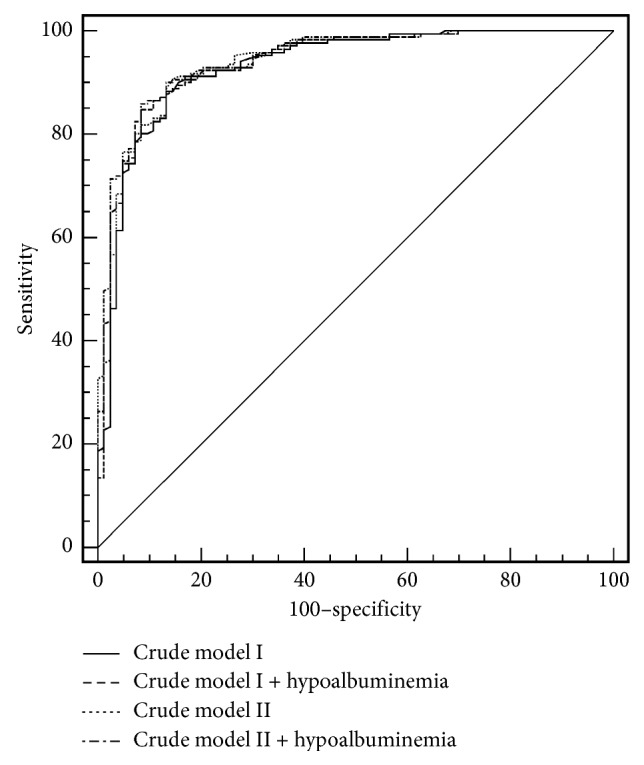
Prognostic value of hypoalbuminemia for the prediction of 6-month poor neurologic outcome. (1) Crude model I (AUC 0.930, 95% CI 0.892–0.958). (2) Crude model I with hypoalbuminemia (AUC 0.938, 95% CI 0.901–0.965). (3) Crude model II (AUC 0.940, 95% CI 0.903–0.966). (4) Crude model II with hypoalbuminemia (AUC 0.944, 95% CI 0.908–0.969).

In receiver operating characteristic (ROC) curve analysis, the area under the curve (AUC) of crude model I was 0.930 (95% CI 0.892–0.958).  Figure 2 shows the AUCs combined with hypoalbuminemia in crude models I and II (AUC 0.938 for crude model I with hypoalbuminemia and 0.944 for crude model II with hypoalbuminemia).

## 4. Discussion

The main finding of this study was that hypoalbuminemia immediately after ROSC was common and was associated with neurologic outcome at 6 months after CA in patients treated with TTM. This association persisted after adjusting for patient-level covariates. However, we could not identify whether correction of hypoalbuminemia improved patient outcome.

Albumin is synthesized in the liver and has a half-life of approximately 3 weeks, and an albumin concentration less than 3.5 g/dl is generally referred to as hypoalbuminemia. Albumin plays important physiologic functions, and hypoalbuminemia is a well-known risk factor for coronary artery disease, acute myocardial infarction, heart failure, stroke, and sepsis [14–17, 22]. In addition, albumin is an indirect indicator of nutritional status. Matsuyama et al. first reported that a higher serum albumin concentration at hospital admission was associated with favorable neurologic outcome after OHCA in a concentration-dependent manner [23]. However, the authors could not analyze the underlying diseases of individual patients (e.g., chronic renal disease) that are commonly associated with hypoalbuminemia.

There are several possible underlying mechanisms explaining the relationship between hypoalbuminemia and poor neurologic outcomes in OHCA patients treated with TTM. First, the most important pathophysiologic mechanism leading to morbidity and mortality after CA is systemic inflammation after ischemic reperfusion injury. Albumin is known as a negative acute-phase protein whose concentration decreases in response to inflammation [10]. Therefore, a low serum-albumin level could be a marker of severe systemic inflammatory responses that could lead to worse outcomes after CA. Second, another possible link between hypoalbuminemia and poor neurologic outcomes in patients with CA treated with TTM is oxidative stress. Albumin constitutes the major plasma protein target of oxidative stress because of reactive oxygen species [24]. Albumin contains a rich thiol group that accounts for 80% of total thiol in plasma-depleting reactive oxygen species [25]. For this reason, reduced serum albumin may be associated with increased oxidative stress conditions. Third, serum albumin is one of the biochemical markers of nutritional status. Serum albumin is closely correlated with the degree of malnutrition, considered a general risk factor in critically ill conditions. Finally, albumin has neuroprotective properties. Experimental studies showed that human albumin administration was highly neuroprotective in reducing infarct volume and cerebral edema in animals with acute stroke [26–28]. Despite the fact that clinical trials have failed to show efficacy of human serum albumin administration after acute ischemic stroke, further studies should be performed to prove the neuroprotective effect of human serum albumin [29].

In our study, the hypoalbuminemia group was older than the normal serum albumin group was and had longer anoxic time, lower incidence of initial shockable rhythm, lower mean arterial blood pressure, and higher incidence of pneumonia than did the normal serum albumin group. Although the exact mechanism could not be explained in this study, it is thought that the abovementioned various hypotheses are a synthesized result. Chronic renal disease, known as an independent risk factor for mortality after CA, was more common in the hypoalbuminemia group. Although we adjusted for chronic renal disease in the multivariate logistic regression model, this factor could affect neurological outcomes after CA with TTM.

This study has several limitations. First, this was a single-center study, which limits the generalizability of our findings. Second, only albumin levels at the time of admission were analyzed, and subsequent albumin levels were not analyzed. In addition, we could not determine whether albumin replacement was performed in the hypalbuminemia group, and we do not know whether albumin replacement improved neurologic prognosis. Third, most patients in our cohort were managed with TTM at 33°C for 24 h regardless of their initial rhythm, and our finding may thus not be applicable to other management strategies.

## 5. Conclusion

Hypoalbuminemia was common after CA, and the serum albumin level at admission was associated with poor neurological outcomes at 6 months after CA in patients treated with TTM. Further studies should be performed to determine whether the correction of hypoalbuminemia improves neurological outcomes.

## Figures and Tables

**Table 1:** Baseline characteristics between patients with and without admission hypoalbuminemia.

	Hypoalbuminemia (<3.5 g/dl, *n* = 109)	Normal serum albumin (≥3.5 g/dl, *n* = 146)	*p*
*Demographics*			
Male	76 (69.7)	104 (71.2)	0.794
Age, years	61.7 ± 15.9	49.5 ± 15.3	<0.001

*Comorbidities*			
CAD	12 (11.0)	19 (13.0)	0.628
CHF	2 (1.8)	6 (4.1)	0.472
Stroke	3 (2.8)	2 (1.4)	0.654
Hypertension	40 (36.7)	36 (24.7)	0.038
Diabetes mellitus	36 (33.0)	19 (13.0)	<0.001
Renal disease	14 (12.8)	3 (2.1)	0.001
Liver disease	2 (1.8)	0 (0.0)	0.182
Malignancy	7 (6.4)	4 (2.7)	0.213

*Resuscitation variables*			
Cardiac cause	71 (65.1)	104 (71.2)	0.299
Shockable rhythm	23 (21.1)	75 (51.4)	<0.001
Witnessed	75 (68.8)	107 (73.8)	0.383
Bystander CPR	56 (51.4)	86 (59.3)	0.207
Anoxic time (min)	37.4 ± 18.3	30.9 ± 20.7	0.009

*Post-ROSC N/Ex*			
PLR	26 (23.9)	69 (47.3)	<0.001
Self-respiration	41 (37.6)	90 (61.6)	<0.001
GCS motor, yes	14 (12.8)	46 (31.5)	<0.001

*Hemodynamic status*			
MAP	83.9 ± 26.9	97.1 ± 25.5	<0.001
HR	100.9 ± 28.3	105.0 ± 26.9	0.234
Temperature	35.3 ± 1.6	35.8 ± 1.0	0.005

*Outcomes*			
Pneumonia	56 (51.4)	53 (36.3)	0.016
Septic shock	10 (9.2)	16 (11.0)	0.641
HD (days)	7.0 (3.0–20.0)	12.0 (6.0–20.0)	0.005
6-month mortality	90 (82.6)	59 (40.4)	<0.001
6-month poor neurologic outcome	95 (87.2)	76 (52.1)	<0.001

CAD, coronary artery disease; CHF, congestive heart failure.

**Table 2:** Comparison of patients according to 6-month neurologic outcome.

	Good neurologic outcome (*n* = 84)	Poor neurologic outcome (*n* = 171)	*p*
*Demographics*			
Male	64 (76.2)	116 (67.8)	0.169
Age, years	48.3 ± 15.2	57.9 ± 16.5	<0.001

*Comorbidities*			
CAD	12 (14.3)	19 (11.1)	0.466
CHF	3 (3.6)	5 (2.9)	0.721
Stroke	0 (0.0)	5 (2.9)	0.175
Hypertension	16 (19.0)	60 (35.1)	0.008
Diabetes mellitus	5 (6.0)	50 (29.2)	<0.001
Renal disease	0 (0.0)	17 (9.9)	0.001
Liver disease	0 (0.0)	2 (1.2)	0.449
Malignancy	1 (1.2)	10 (5.8)	0.074

*Resuscitation variables*			
Cardiac cause	79 (94.0)	96 (56.1)	<0.001
Shockable rhythm	68 (81.0)	30 (17.5)	<0.001
Witnessed	72 (85.7)	110 (64.7)	<0.001
Bystander CPR	52 (61.9)	90 (52.9)	0.176
Anoxic time (min)	23.6 ± 15.0	38.6 ± 20.3	<0.001

*Post-ROSC N/Ex*			
PLR	64 (76.2)	31 (18.1)	<0.001
Self-respiration	73 (86.9)	58 (33.9)	<0.001
GCS motor, yes	47 (56.0)	13 (7.6)	<0.001

*Hemodynamic status*			
MAP	99.2 ± 25.2	87.6 ± 26.9	0.001
HR	101.2 ± 25.7	104.3 ± 28.4	0.407
Temperature	35.8 ± 0.9	35.5 ± 1.4	0.060
Hypoalbuminemia	14 (16.7)	95 (55.6)	<0.001
Albumin (mg/dl)	3.9 ± 0.4	3.3 ± 0.6	<0.001

*Outcomes*			
Pneumonia	26 (31.0)	83 (48.5)	0.008
Septic shock	4 (4.8)	22 (12.9)	0.049
HD (days)	7 (4–20)	15 (9–23)	<0.001

**Table 3:** Odds ratios for hypoalbuminemia in the prediction of 6-month poor neurologic outcome.

	OR (95% CI)	*p*	Adjusted OR (95% CI)	*p*
*Hypoalbuminemia*	6.250 (3.268–11.951)	<0.001		
Crude model I			3.526 (1.388–8.956)	0.008
Crude model II			2.658 (1.017–6.949)	0.046

*Serum albumin* (mg/dl)	0.138 (0.073–0.262)	<0.001		
Crude model I			0.222 (0.095–0.516)	<0.001
Crude model II			0.275 (0.116–0.651)	0.003

Crude model I: cardiac etiology + initial rhythm + anoxic time + GCS motor score. Crude model II: crude model I + liver disease + kidney disease + malignancy.

## Data Availability

The data used to support the findings of this study are available from the corresponding author upon request.
